# CUSUM charts in the quality control of colon cancer lymph node analysis: a population-registry study

**DOI:** 10.1186/s12957-018-1533-0

**Published:** 2018-11-30

**Authors:** Carlos Fortea-Sanchis, David Martínez-Ramos, Javier Escrig-Sos

**Affiliations:** 1 Department of Surgery, Division of Colorectal Surgery, Consorcio Hospitalario Provincial de Castellón, Av. Doctor Clara, 19, 12002 Castellón, Spain; 2 Department of Surgery, Hospital General de Castellón, Av. Benicassim s/n, 12004 Castellón, Spain

**Keywords:** Colon cancer, Lymph nodes, Survival, Quality control

## Abstract

**Background:**

The most important determinant of survival in patients with colon cancer is the presence or absence of regional lymph node metastases. This factor is consistently associated with long-term and disease-specific survival. Cumulative summation of differences (CUSUM) charts can help to discriminate abnormalities that cannot be explained by the general variability of a process. We used CUSUM charts to analyse the quality of nodal analysis in colon cancer and to use a population-registry cancer database to estimate the optimal number of lymph nodes for adequate prognostic analysis.

**Methods:**

This was a multicentre population-registry cancer study from January 2004 to December 2007. We used these data to produce the different CUSUM curves, focusing on the main variables. To calculate survival, we used the Kaplan–Meier method.

**Results:**

In this study, we examined 548 patients. The CUSUM curves were calculated for overall mortality, specific mortality, and recurrence according to (1) the number of lymph nodes analysed and affected and (2) compared the ratio of the number of lymph nodes affected to the number analysed. Finally, the lymph node ratio was compared to the overall survival CUSUM curve.

**Discussion:**

This CUSUM control chart analysis reinforces the unquestionable importance of analysing at least 12 lymph nodes in patients with colon cancer in order to accurately estimate their prognosis. However, our findings indicate that the analysis of at least 20 lymph nodes is a more appropriate cutoff point for accomplishing the demanding objective of diagnosing a high-quality prognosis in colon cancer patients.

## Background

Colorectal cancer is the third most common cancer worldwide and is the most frequent malignancy in many Western countries. Approximately 2359 new cases are diagnosed per 100,000 individuals each year, and it is the second leading cause of death from cancer both in men and women in Spain (after lung and breast cancer, respectively) [1]. After distant metastases, the second most important determinant of survival among patients with colon cancer is the presence or absence of regional lymph node metastases. This factor is consistently associated with long-term and disease-specific survival [2, 3], and the presence of these metastases has important implications because it may determine the use of adjuvant therapies [2, 4]. Furthermore, the total number of lymph nodes evaluated (even those negative for metastasis) is consistently associated with disease-specific survival in patients with stage II and III cancer as well as with long-term disease survival [2, 3, 5].

The recommended number of lymph nodes for analysis in colon cancer ranges from 6 to more than 30 nodes and has been repeatedly scrutinised in the literature using different statistical methods in an attempt to identify an optimal cutoff number [6, 7]. Generally, 12 is considered the gold standard for colon cancer, although no studies have specifically demonstrated that this number is the most advantageous. Moreover, using classical statistical methods, it is difficult to discriminate specific differences presented by any given procedure from their general variability or any of their other variables.

Cumulative summation of differences (CUSUM) charts are used in quality control for industrial processes because they can help to discriminate abnormalities that cannot be explained by the general variability of a process. In clinical care processes, this property can be applied to identify which sections of an outcome influence a variable that can affect the result. Therefore, CUSUM charts are also useful for assessing the learning curve and for more generally assessing quality-of-care results [8–11]. However, this analysis system has not yet been tested for lymph node analyses in colorectal cancer. Thus, here, we used CUSUM charts to analyse the quality of nodal analysis in colon cancer and to use a population-registry cancer database to estimate the optimal number of lymph nodes for adequate prognostic analysis.

## Methods

This was a multicentre population-registry cancer study; data from this registry are included in the EUROCARE study [12]. The study period was from January 2004 to December 2007, and the inclusion and exclusion criteria are shown in Table 1. The variables used were age, gender, location, size, histology, grade of differentiation and tumour extension, number of lymph nodes analysed, number of lymph nodes affected, lymph node ratio (LNR)—i.e. the ratio of affected lymph nodes to those analysed, year of diagnosis, specific and overall survival, date of metastasis or recurrence, follow-up time, and TNM stage and condensed TNM—both according to the sixth edition of the Union for International Cancer Control (UICC).

**Table 1:** Inclusion and exclusion criteria

Inclusion criteria	Exclusion criteria
Surgery with curative intent with lymph node resection	Palliative surgery without lymphadenectomy
Full pathology report	Incomplete pathology report
Clear clinical status at last follow-up	Doubtful clinical status at the last follow-up
Colon tumours	Appendiceal and rectal tumours
	Surgery without resection
	Metastasis at diagnosis
	Inadequate follow-up

CUSUM graphs were used to distinguish different groups within continuous-type prognostic variables following the method described by Barrio et al. [13]. To identify the cutoff, we calculated the predicted probabilities using logistic regression for a binary variable resulting from a continuous prognostic variable. The thresholds are determined by the CUSUM graphs we used to monitor the trend changes in the probabilities calculated by this type of logistic regression, as well as these changes in trend themselves [14].

We used these data to produce the different CUSUM curves, focusing on the main variables. First, we calculated a CUSUM curve for overall mortality according to the number of lymph nodes analysed and affected. Second, we compared specific mortality with the number of lymph nodes analysed and affected. Finally, we used the CUSUM chart to calculate predicted overall survival according to the LNR. To calculate survival, we used the Kaplan–Meier method. All our analyses and the generation of the CUSUM graphs were carried out using SPSS, version 17.0, for Windows.

## Results

During this 4-year study, 944 patients were diagnosed with colon cancer; 279 did not fulfil the inclusion criteria and a further 116 cases with metastases at diagnosis were also excluded. Thus, a total of 548 patients were examined in this study; their main epidemiological and tumour characteristics are summarised in Table 2. A total of 6400 lymph nodes were analysed (median 10 nodes per case; range 1–45). Fewer than 12 lymph nodes were analysed in 310 patients (56.3%) while 12 or more lymph nodes were checked in 241 cases (43.7%). The median follow-up time was 51 months (range 0–99 months). During follow-up, metastases appeared in 92 cases (16.7%), there was a local recurrence in 39 cases (7.1%), and 214 patients died (38.8%). According to our Kaplan–Meier analysis, the 5- and 10-year overall survival was 89% and 79%, respectively. The 5-year disease-specific survival rates were 95% and 88% at 10 years, and the 5-year disease-free survival (without metastases or recurrences) was 90% and 81% at 10 years.

**Table 2:** Epidemiological and tumour characteristics

	*n* = 548
Age*	72 (63–80)
Grouped age	109 (19.9%)
< 60	222 (40.5%)
> 75	217 (39.6%)
Gender	
Female	252 (46%)
Male	296 (54%)
Tumour location	
Right colon	195 (35.6%)
Transverse colon	58 (10.6%)
Left colon	44 (8.0%)
Sigmoid colon	217 (39.6%)
Unknown	34 (6.2%)
Histology	
Adenocarcinoma	461 (84.7%)
Mucinous	74 (13.6%)
Signet-ring cell	9 (1.7%)
Major size (mm)*	45 (32–55)
Grade	
Unknown	19 (3.5%)
I	151 (27.6%)
II	343 (62.6%)
III	35 (6.4%)
Adjuvant chemotherapy	
No	371 (67.7%)
Yes	177 (32.3%)
Number of retrieved lymph nodes*	10 (7–15)
Cutoff retrieved lymph nodes	
< 12	308 (56.2%)
≥ 12	240 (43.8%)
Number of positive lymph nodes*	0 (0–1)
Lymph node ratio	
0–24	448 (81.8%)
25–60	80 (14.6%)
> 60	20 (3.6%)
Condensed pT6	
T1–T2	118 (21.5%)
T3–T4	430 (78.5%)
Condensed pN7	
N0	346 (63.1%)
N1	143 (26.1%)
N2	59 (10.8%)
Condensed TNM stage	
I	93 (17%)
II	253 (46.2%)
III	202 (36.8%)
Postoperative death (90 days)	
No	503 (91.8%)
Yes	45 (8.2%)
Follow-up general mortality	
No	334 (60.9%)
Yes	214 (39.1%)
Follow-up recurrence	
No	439 (80.1%)
Yes	109 (19.9%)
Follow-up time (months)*	51 (30–64)

*Median (IQR: interquartile range)

**Fig. 1:**
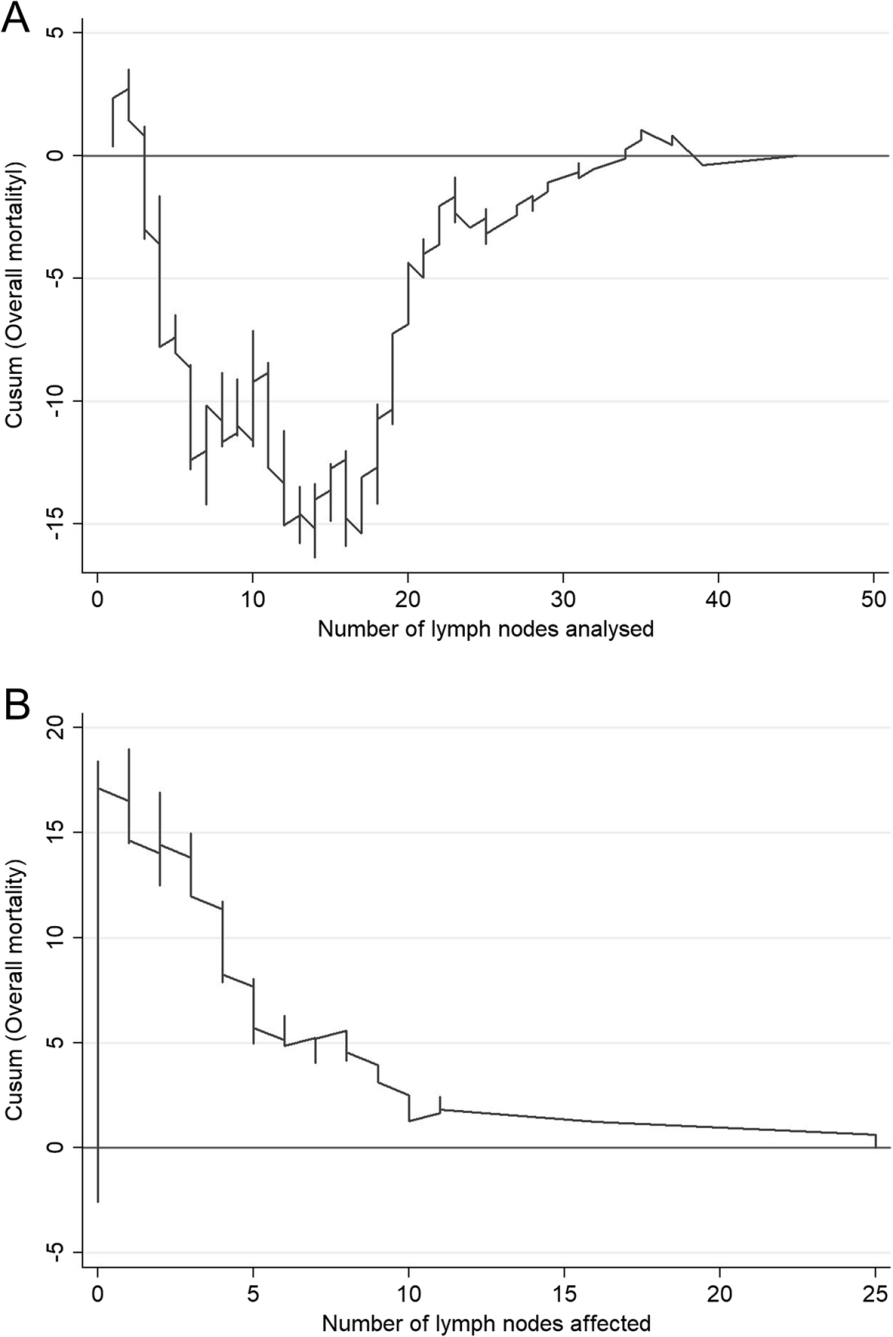
CUSUM curves. **a** Overall mortality according to the number of lymph nodes analysed. **b** Overall mortality according to the number of lymph nodes affected

The CUSUM curve for overall mortality was calculated according to the number of lymph nodes analysed (Fig. 1a). This graph shows that the risk of mortality initially tended to increase (downward trend) until approximately 12 nodes were analysed; after this, there was a trend of falling risk (upward curve) until approximately 20 nodes were analysed; at this point, the risk slowly increased (the curve rises slowly). These trend changes translate into a significant increase in the probability of death when fewer than 12 lymph nodes were analysed. From a practical standpoint, these results indicate that analysing fewer than 12 lymph nodes favours worse outcomes; the intensity of this correlation subsequently decreases (but is not null) and stabilises at around 20 nodes (Fig. 1a). Consequently, producing an accurate prognostic diagnosis in patients with colon cancer requires the retrieval of at least 20 lymph nodes (and never fewer than 12).

**Fig. 2:**
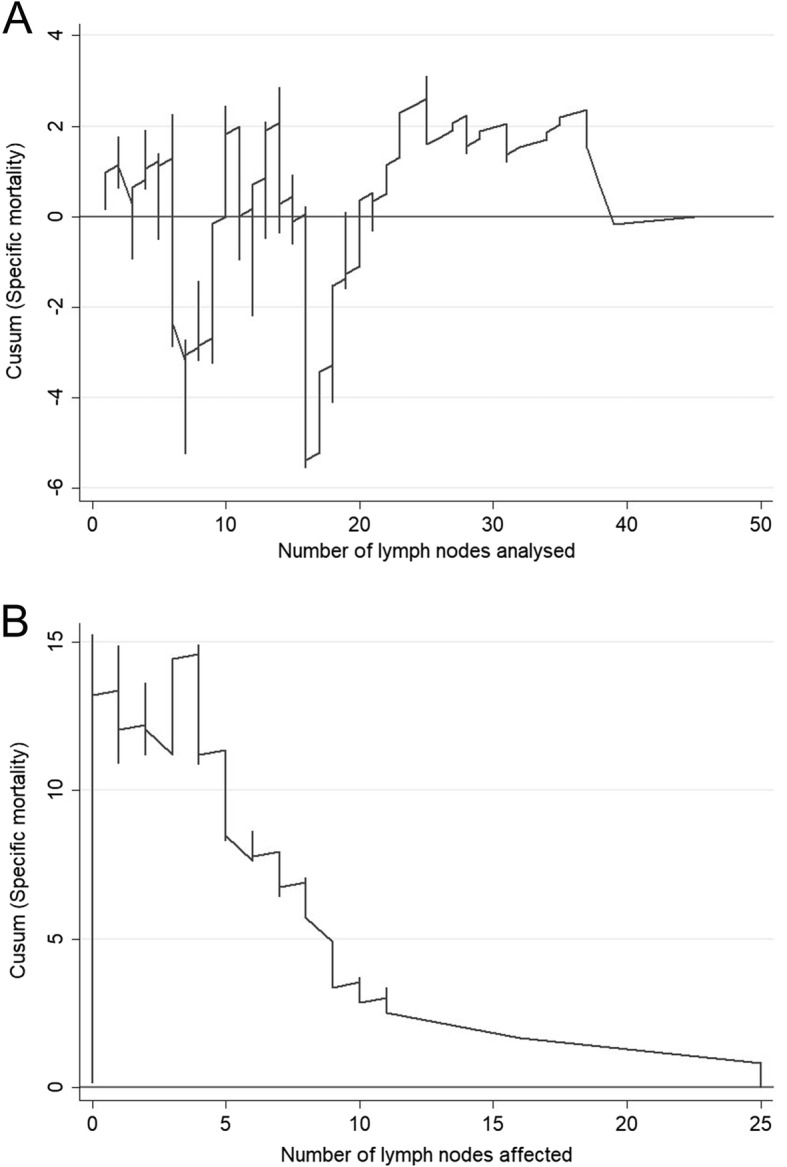
CUSUM curves. **a** Specific mortality according to the number of lymph nodes analysed. **b** Specific mortality according to the number of lymph nodes affected

**Fig. 3:**
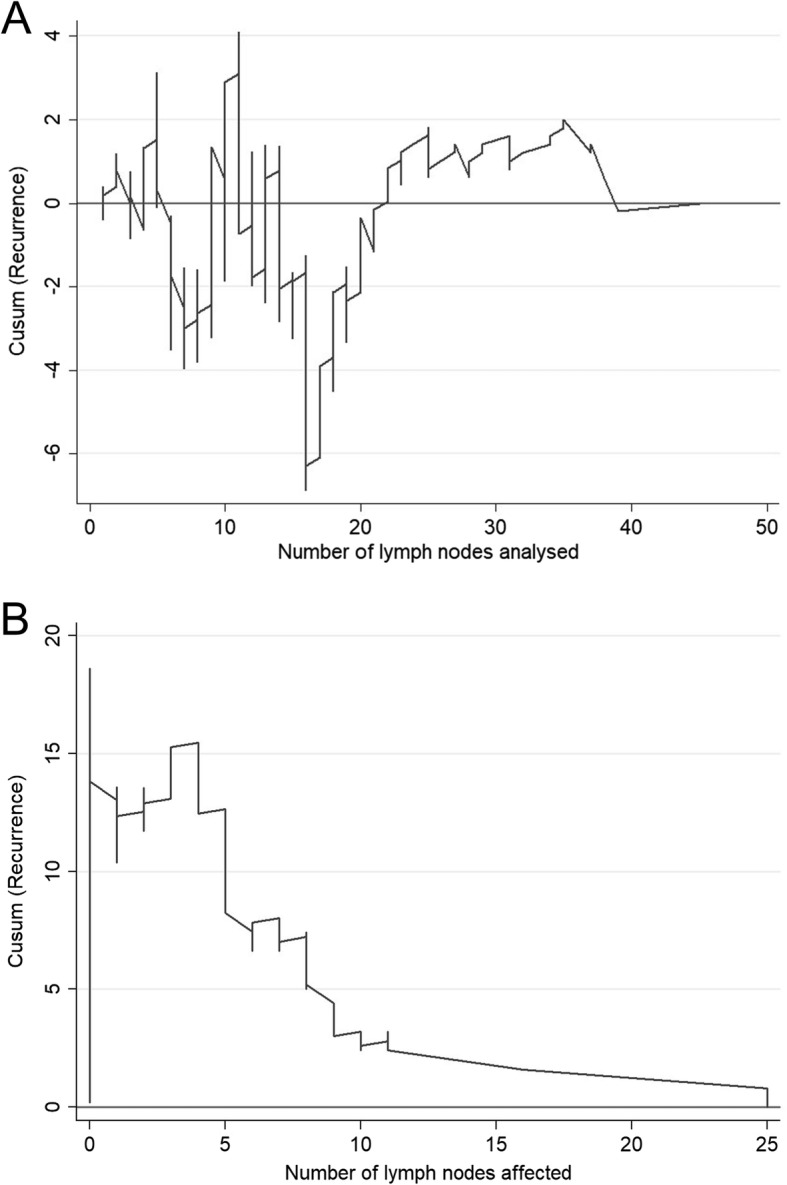
CUSUM curves. **a** Recurrence according to the number of lymph nodes analysed. **b** Recurrence according to the number of lymph nodes affected

Figure 1b compares the general mortality to the number of lymph nodes affected and shows that the risk of mortality tends to increase in line with the number of affected lymph nodes (between 1 to 10 affected lymph nodes), as seen as in the area where the curve descends; after this point, the trend stabilises. As predicted, the risk of mortality increases as a function of the number of lymph nodes affected. A comparison of specific mortality versus the number of lymph nodes analysed initially shows high fluctuation above and below the null line, but a strong trend of decreasing specific mortality clearly emerges after 21 lymph nodes are analysed (Fig. 2a). The CUSUM graph analysis of the specific mortality according to the number of affected nodes (Fig. 2b) showed a marked increase in the mortality risk trend when between 1 and about 10 affected lymph nodes were found (downward curve), followed by a slower trend of increasing mortality risk. This means that the differential mortality risk increased linearly as more positive nodes were analysed. As in its counterpart graph for mortality (Fig. 2a), there is a clear relationship between the number of affected lymph nodes and recurrence (Fig. 3a), even when many lymph nodes were analysed (Fig. 3b).

**Fig. 4:**
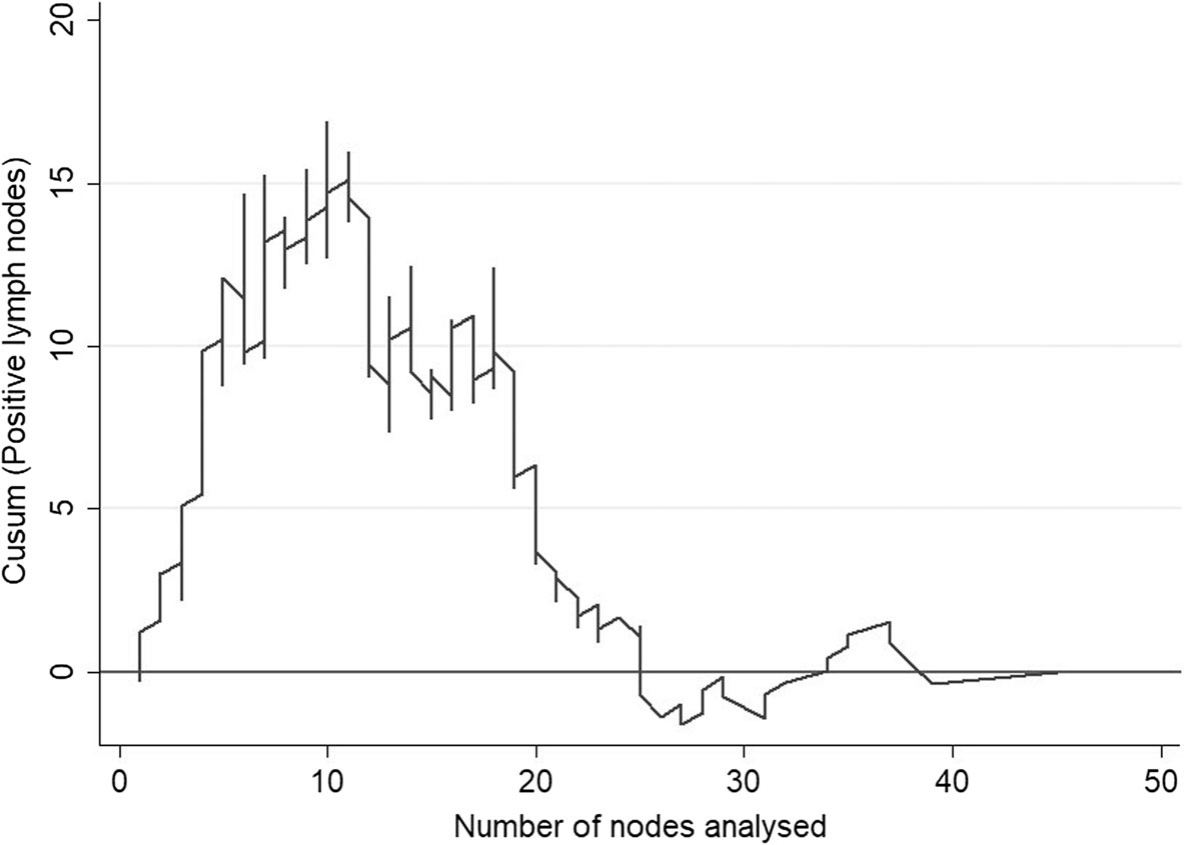
CUSUM curve. Positive lymph nodes according to the number of nodes analysed

**Fig. 5:**
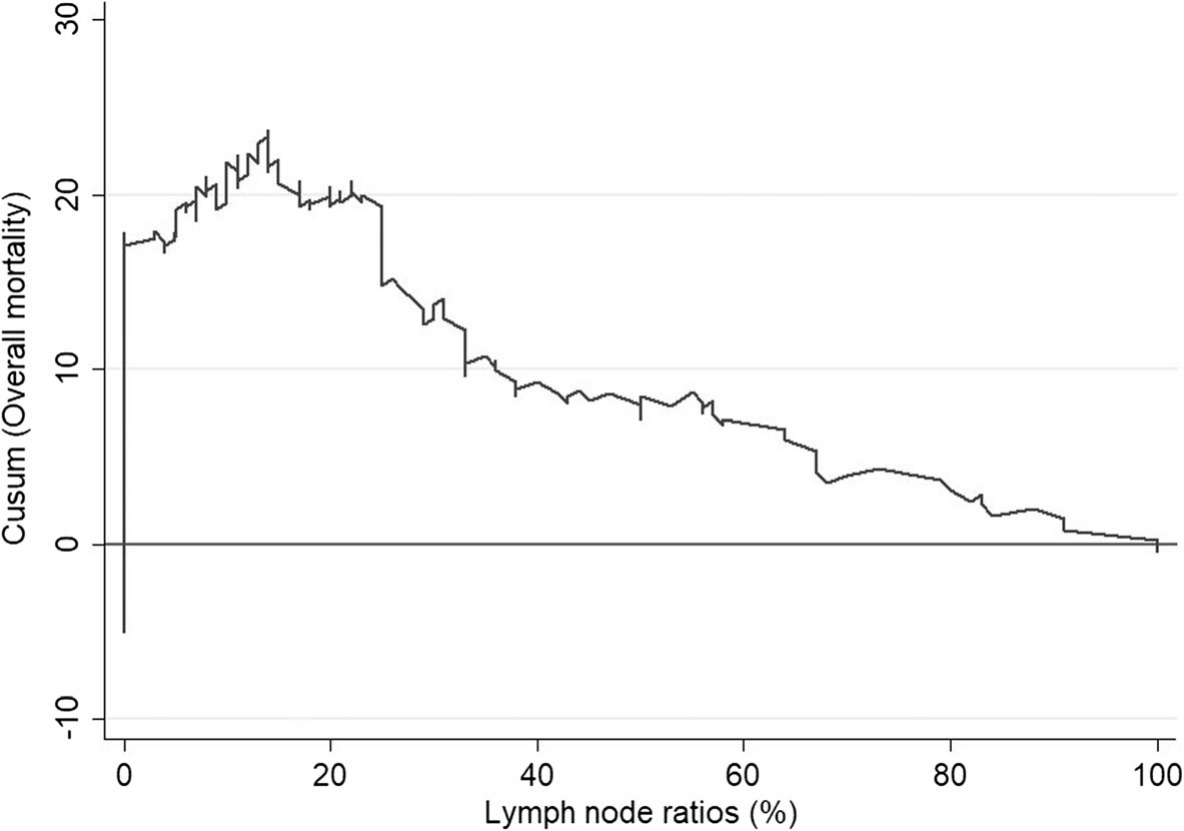
Overall survival according to the lymph node ratios (LNRs)

**Fig. 6:**
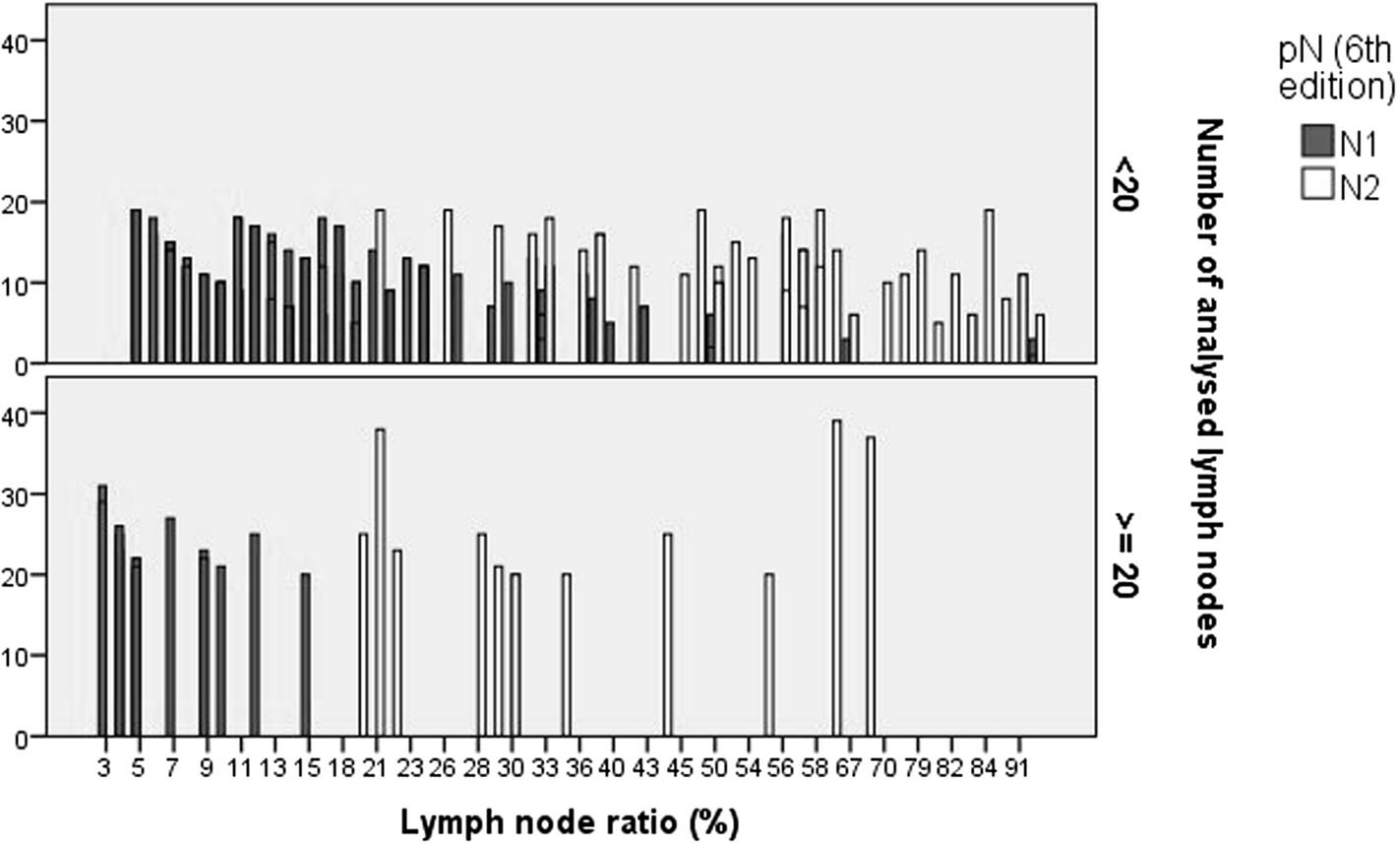
Lymph node ratios versus pN classifications

The CUSUM curve comparing the ratio of the number of lymph nodes affected by the number analysed (Fig. 4) highlights two clear trends: the odds of finding affected lymph nodes decreases until 10–11 lymph nodes are analysed, after which the probability increases to stabilise at 23–24 nodes, and there is no clear increase in the probability of finding more positive nodes. Finally, according to the LNR versus overall survival CUSUM curve, the risk of death clearly and consistently increases when the LNR was 20% or more (Fig. 5). When LNR was compared with the pN category (Fig. 6), the pN1 and pN2 categorisation perfectly matched the 20% LNR when high-quality nodal analysis (i.e. on more than 20 lymph nodes) was carried out.

## Discussion

Given the importance of lymph node analysis in colon cancer, many studies have been conducted in this field [2–5]; however, this is the first time CUSUM charts have been used for this type of assessment. CUSUM charts are very useful for detecting subtle changes in the trend of any process [10], and their use is becoming more widespread in medical fields [11], especially in the study of learning curves [15, 16]. Hence, here, we explored their usefulness in the analysis of lymph node status in colon cancer. CUSUM charts are based on sequentially monitoring cumulative performance over time to display the trends in the relationship between two variables: a qualitative, binary, result variable (*Y* axis) and a quantitative variable which can predict this result (*X* axis). Thus, the horizontal axis represents the cases over time and, bellow the null line, the vertical axis indicates the lives saved compared to the number of expected saved lives [14].

Following a meeting of experts in Sydney in 1991, the international consensus on the minimum number of lymph nodes that should be analysed to be able to correctly stage colon cancer was set at 12, and the international community subsequently adopted this number as optimal [17, 18]. The data that we present here clearly agree that the minimum number of nodes required for an accurate prognostic diagnosis in these patients is 12 (Fig. 1a, b). However, we also show that collecting many more lymph nodes (20 or more) would be advisable because an increased risk of mortality persists when fewer are collected. This may be because the disease stage may be underestimated in patients with fewer analysed lymph nodes. Coinciding with our results, many recent studies have questioned the use of this number of lymph nodes [2] and recommend retrieving as many nodes as possible [3, 19].

In this sense, identification of a significant cutoff number of 20 lymph nodes in this study is of great importance, both in terms of overall survival (Fig. 1a) and in other variables, including mortality (Fig. 2a) and recurrences (Fig. 3a). Moreover, as more positive lymph nodes are found, overall mortality (Fig. 1b), specific mortality (Fig. 2b), and recurrence (Fig. 3b) also increase; thus, the more lymph nodes affected, the higher the risk. The use of LNR—the ratio between affected and analysed lymph nodes—has been previously reported in several studies which consider it a prognostic factor more important than the specific number of nodes analysed [5, 20]. Furthermore, LNR can also be used in cases where data for the recommended minimum number of lymph nodes are not available—as in more than 50% of the patients included in the population registry we used in this study. As shown in Fig. 6, the LNR is equivalent to the pN in cases with high-quality nodal analysis (more than 20 lymph nodes analysed). Therefore, CUSUM charts appear to be most useful for choosing the best discriminative cutoff nodal ratio for survival prognosis in different cancer types.

Using the aforementioned statistical methods, CUSUM curves can be used to identify the groups that best discriminate the prognosis on the basis of a given result variable. Categorising highly discriminant prognostic variables in this way will always maximally stratify the main outcome in randomised clinical trials or, for non-randomised trials, will produce the best adjustment of the confounding factors. This is a universal rule of all statistical analyses and helps us to discover the best treatment for various patient groups.

In summary, our analysis with CUSUM control charts reinforces the unquestionable importance of analysing at least 12 lymph nodes in patients with colon cancer in order to accurately estimate their prognosis. However, our results highlight the fact that 12 nodes must be the minimum number and that 20 or more nodes should be analysed to obtain the most useful and highest quality information. Our findings indicate that the analysis of at least 20 lymph nodes is a more appropriate cutoff for accomplishing the demanding objective of the high-quality diagnosis of prognosis in colon cancer patients.

## Conclusions

Twelve nodes must be the minimum number analysed in colon cancer to accurately estimate patient prognosis. However, the analysis of at least 20 lymph nodes is a more appropriate cutoff for accomplishing the demanding objective of the high-quality diagnosis of prognosis.

## Abbreviations

CUSUM: Cumulative summation of differences
LNR: Lymph node ratios
UICC: Union for International Cancer Control
